# Respiratory syncytial virus: A review of current basic and clinical knowledge

**DOI:** 10.5339/qmj.2024.56

**Published:** 2024-12-24

**Authors:** Obada Al-leimon, Hani Shihadeh, Ahmad Ayman Yousef, Anas Khraim, Rizeq Siwwad

**Affiliations:** 1 School of Medicine, The University of Jordan, Amman, Jordan *Email: Obada-nayef@outlook.com

**Keywords:** Respiratory syncytial virus, pneumonia, RSV vaccine, Nirsevimab

## Abstract

**Background:**

Respiratory syncytial virus (RSV) is a highly contagious pathogen known for causing respiratory tract infections, particularly among pediatric and elderly patients. Its ability to induce outbreaks in both community and hospital settings underscores its substantial health burden. This review aims to provide a comprehensive understanding of RSV, including its biological and clinical aspects.

**Methods:**

A comprehensive review of the literature was conducted by searching PubMed, Scopus, and Web of Science databases for relevant articles. Key topics included RSV virology, epidemiology, clinical findings, diagnostic methods, management approaches, and preventive strategies.

**Results:**

This review encompasses the taxonomy and structure of RSV, including its genome and proteins. Various strains and their dominance patterns, alongside pathogenesis mechanisms, are explored. Diagnostic techniques such as nucleic acid amplification tests are discussed for their efficacy and accessibility. Supportive care remains the primary treatment, with antiviral therapies playing a limited role. Monoclonal antibody immunization and vaccination efforts offer promising avenues for RSV prevention. The impact of the COVID-19 pandemic on RSV epidemiology is also considered, along with the oncolytic potential of RSV in cancer treatment.

**Conclusion:**

Advancements in understanding RSV virology, epidemiology, and clinical management have paved the way for improved diagnostic and preventive strategies. However, challenges remain in ensuring widespread access to diagnostics and effective treatments, particularly in resource-limited settings. Continued research and global collaboration are essential for addressing the ongoing impact of RSV and reducing its burden on public health.

## 1. Introduction

Respiratory syncytial virus (RSV) is a significant global respiratory pathogen, particularly affecting vulnerable populations like infants, young children, and older adults. It causes a spectrum of respiratory illnesses, ranging from mild upper respiratory infections to severe bronchiolitis and pneumonia.^1^ According to the centers for disease control and prevention (CDC), in the United States, children under 5 years old experience approximately 2.1 million outpatient visits and 58,000–80,000 hospitalizations, while adults aged 65 and older face 60,000–160,000 hospitalizations per year. The typical seasonal pattern involves RSV season starting in fall, peaking in winter, and declining in spring. However, the COVID-19 pandemic disrupted these patterns.^2^

Studies from the 1990s revealed that multiple RSV genotypes circulate during outbreaks, with one or two predominating before declining. Genotypes show worldwide distribution and temporal, not geographic, circulation. Antigenic variation contributes to high reinfection rates. Both RSV groups A and B have circulated concurrently for over 20 years globally, with varying prevalence and pathogenicity.^3^

This review thoroughly examines RSV, covering its viral classification, structure, disease process, transmission patterns, clinical presentations, diagnostic methods, treatment approaches, preventative strategies, and recent developments. In 2019, RSV caused 33 million severe lower respiratory tract infections, 3.6 million of which required admission, and it caused nearly 100,000 deaths.^4^ Hence, understanding RSV and having basic knowledge of its diagnosis and management is crucial for most physicians, especially pediatricians.

Our goal is to enhance the collective knowledge surrounding RSV, supporting informed decision-making among healthcare professionals, researchers, and policymakers. By synthesizing current knowledge and advancements, we offer insights into the challenges posed by RSV and explore potential strategies for reducing its public health impact.

## 2. Methods

This manuscript summarizes results from previously published data on RSV’s virology, including taxonomy, genome, and proteome; pathogenesis including steps of viral replication and rules of different viral proteins; epidemiology, pathogenesis, laboratory, radiologic, and clinical diagnosis; management, prevention; and RSV as an oncolytic virus. We searched in PubMed, Scopus, and Web of Science databases. We utilized snowballing to identify other relevant articles. Only peer-reviewed articles in English, published after 2010, and their full text available were considered and we chose the most relevant sources. We retrieved 7751 articles; 103 articles were found to be relevant.

## 3. The Pathogen

### 3.1. Taxonomy and virion structure

RSV belongs to the Orthopneumovirus genus of the family Pneumoviridae (recently classified as a separate family from Paramyxoviridae) of the order Mononegavirales.^5^

RSV virion is composed of an envelope and a helical nucleocapsid core consisting of a negative-sense single-stranded ribonucleic acid (RNA) genome and three proteins: nucleocapsid (N), large (L), and phosphor (P) proteins. The envelope contains three types of glycoproteins: glycoprotein (G protein), which is necessary for viral attachment, fusion protein (F protein), which facilitates infected cells’ fusion and formation of syncytia (hence the name), and small hydrophobic protein (SH protein), which functions as an ion channel. F and G proteins are essential for viral attachment and replication, and neutralizing antibodies against these proteins protect against infections. Hence, they are essential for vaccine development.^5-7^

### 3.2. Pathogen genome and proteins

RSV genome consists of 10 genes that encode 11 proteins: nine structural and two nonstructural. Structural proteins include three external envelope proteins: receptor attachment G, F, and SH proteins.^6-10^ The genome also encodes the N, which forms a complex with P and L to form an RNA-dependent RNA polymerase complex, which is responsible for both transcription and replication of viral RNA, and thus it can provide a valuable antiviral drug target.

M2-1 and M2-2 proteins are produced from the same viral gene (M2) from two different open reading frames. M2-1 functions as a processivity factor for viral RNA polymerase, thus enhancing the synthesis of viral RNA. M2-2 is involved in switching viral RNA synthesis from transcription, which involves the production of subgenomic +ssRNA segments that are translated into viral proteins, into replication, which involves the production of a genomic +ssRNA that serves as a template for the synthesis of genomic -ssRNA.^11^ Nonstructural proteins 1 and 2 (NS-1 and NS-2) help the virus evade the immune response by interfering with interferon signaling.^5,11–13^

### 3.3. Strains of RSV and their dominance

Multiple genotypes of RSV exist, usually defined by the G gene sequence that has the most variability in the RSV genome. These genotypes are classified into two large groups: RSV-A and RSV-B. The presence of many genotypes is thought to be responsible for the ability of the RSV to cause reinfection.^14,15^ Because of this variability in the G protein, most RSV therapeutic vaccines and antibodies target the F protein, which is more preserved among different genotypes.

There are three types of RSV G protein epitopes: (1) conserved epitopes, found in all RSV genotypes; (2) group-specific epitopes, shared only by genotypes within the same group; and (3) genotype-specific epitopes that define a certain genotype of RSV.^14,16^

Gene sequencing analysis of the G gene identified the presence of 11 RSV-A genotypes (GA1-GA7, NA1, NA2, SAA1, and ON1) and 23 RSV-B genotypes (GB1-GB4, SAB1-SAB4, URU1, URU2, BA1-BA12, and THB).^11,15,17–20^

RSV genotype predominance is complex as the predominant genotype differs between seasons and countries.^10^ However, some studies reported that RSV-A group genotypes are more common than RSV-B group genotypes; of all the genotypes of the RSV-A group, the subtype NA1 was the most frequently observed until the newly identified ON1 subtype was discovered in 2010 in some countries, and now it is becoming more frequent replacing NA1. Also, the BA9 subtype of RSV-B is the most predominant within this group. This dominance of BA9 and ON1 is suggested to be related to the highest structural variation of the G protein, which gives these genotypes the advantage of escaping herd immunity. In contrast, herd immunity may be common against other genotypes.^10,13,16,21,22^

## 4. Pathogenesis

RSV typically enters the host through respiratory droplets from infected persons or contaminated surfaces.^23^ F protein and the G protein of the envelope are responsible for the virus’s attachment to respiratory epithelial cells. There are numerous candidate cellular receptors described for RSV entry, including Annexin II, CX3 chemokine receptor 1 (CX3CR1), epidermal growth factor (EGF) receptor, calcium-dependent lectins, toll-like receptor 4 (TLR4), intercellular adhesion molecule 1 (ICAM-1), nucleolin-1, and heparan sulfate proteoglycans (HSPGs).^7,24^ The interaction between RSV G protein and CX3CR1 seems to be crucial for viral particle attachment to the cell surface as well as for subsequent signaling events. RSV F protein also binds TLR4, expressed on ciliated bronchial epithelial cells in the airway; this binding initiates specific signaling pathways that promote viral entry by increasing virion’s endocytosis. However, TLR4 is not an entry receptor by itself and is not necessary for RSV entry. Then fusion occurs, and the viral nucleocapsid enters the host cell, where it is replicated. RSV-infected cells exhibit characteristic inclusion bodies when seen under an electron microscope.^7^

Many components of the host’s innate immune system are activated as an initial line of defense in response to viral infection.^25,26^ Viral particles and virus-derived nucleic acids are sensed by pattern recognition receptors (PRRs), which then trigger signaling pathways that stimulate the synthesis and secretion of cytokines and chemokines. The most critical cytokines are interferons, which bind to their receptors and activate the Janus kinases, signal transducer, and activator of transcription proteins (JAK/STAT) pathway, which then induces the expression of several IFN-stimulated genes (ISGs). Several well-characterized ISGs whose products exhibit broad and enzymatic antiviral activity include the 2′-5′ oligo adenylate synthetase (OAS)/RNAse L system, which cleaves viral RNA; protein kinase R (PKR), which acts to inhibit viral messenger ribonucleic acid (mRNA) translation; interferon-induced protein with tetratricopeptide repeats 1; and interferon-induced GTP-binding protein Mx1. The interferon response is essential in determining the outcome of RSV infection, which can range from asymptomatic infection to severe acute clinical disease.^25,26^

Chemokines secreted at the site of infection attract innate immune cells, including monocytes, granulocytes, neutrophils, dendritic cells (DCs), natural killer (NK) cells, and macrophages. These professional phagocytes can quickly eliminate virus particles. Activated macrophages produce inflammatory cytokines like TNF-α and IFN-γ that activate the adaptive immune response against RSV. They also present viral antigens to T lymphocytes.^25,26^

NK cells can identify cells infected with RSV without needing a viral antigen presented on major histocompatibility complex class I and then use their cytotoxic properties to eliminate them. Additionally, they secrete interferon-γ, which helps to activate the adaptive immune system.^26^

## 5. Epidemiology (Disease Burden)

RSV is the most common cause of serious, life-threatening respiratory tract infections in children under the age of 5. It is also a significant cause of pneumonia and severe lower respiratory tract infections in the elderly.^24^ The peak incidence of RSV infection occurs between two and three months of age, while most mortalities occur in those younger than six months. Children with some genetic and chromosomal abnormalities (e.g., Down syndrome), pulmonary chronic diseases, cancer, congenital heart disease, premature infants, infants small for gestational age (>2.5 kg), and immunocompromised children are at higher risk of developing severe RSV infections. Male babies are more likely to develop severe RSV infection than female babies.^24,27^

According to the most recent statistics, there are 33 million cases of RSV infection occurring worldwide in children under five years; 6.6 million of these occur in infants younger than 6 months, and around 10% of cases require hospitalization. RSV causes nearly 100,000 deaths yearly; 97% of all RSV deaths worldwide occur in low-income countries.^4^

## 6. Clinical Features

RSV is recognized as the primary causing of bronchiolitis in infants population.^28^ It may also cause pneumonia, upper respiratory tract infections (URTIs), and acute otitis media.^1,29,30^ Clinical manifestations of RSV infection manifest across a spectrum; they commonly include cough, fever, and runny nose.^29-31^ Affected individuals may also experience diminished appetite.^30,32^ Physical findings of children with RSV infection commonly include expiratory wheezing^33^ and signs indicative of respiratory distress, including chest in-drawing, tachypnea, and crepitations.^34^ Less frequently observed signs include nasal flaring, grunting, accessory muscle usage, and fine rales.^34-36^ Severe cases may experience apnea, hypoxia, altered mental status, and hypotension.^31,37^

Younger infants have higher odds of hospitalization and the requirement for mechanical ventilation (MV) in comparison to RSV-positive infants in the emergency department and outpatient setting.^38,39^ Prematurity, other chronic comorbidities, and thrombocytosis were associated with prolonged hospital stay and a higher risk of pediatric intensive care unit (PICU) admission and mortality.^40^

In healthy term-born children, non-infection with RSV in the first year of life was associated with a reduced risk of developing childhood asthma. The association between RSV infection during infancy and childhood asthma demonstrated age-dependency, emphasizing the need for further studies to establish causality.^41^

RSV can also impact adults, especially the elderly and young adults with compromised immune systems. A study identified 86 hospitalized adult patients with a median age of 74 years. The majority had underlying chronic medical conditions, and common symptoms included cough, shortness of breath, and sputum production. Complications such as respiratory failure and cardiovascular collapse were observed, with a notable portion requiring intensive care.^42^ Another study reported RSV detection in 11% of hospitalized adults, with profound outcomes associated with advanced age and underlying cardiopulmonary disease.^43,44^

### 6.1. Viral genotypes and severity of infection

Researchers wondered whether infection with different genotypes of RSV is related to the clinical severity of infection; contradictory results were found, with some studies reporting that infection with genotypes belonging to RSV-A group causing more severe disease,^45,46^ others reporting that infection with genotypes belonging to RSV-B group causes more severe infection,^47^ and others found no significant difference in severity between infections with genotypes belonging to different groups.^21,46,48,49^ A study found that infants with RSV-A genotype are more likely to be hospitalized, while those with RSV-B genotypes infection have prolonged length of stay as well as a higher risk of PICU admission.^21^ Possible reasons for these contradictory findings may be related to differences in study design, the epidemic strains, the patient’s ethnicity, or previous infection with the pathogen.^45,48^

## 7. Laboratory and Radiological Diagnosis

Once considered the gold standard for identifying respiratory viruses, cell culture has mainly been replaced by more sensitive and less time-consuming methods that depend on detecting viral antigens, viral RNA, DNA, or antibodies specific to the virus.^50^

Nucleic acid amplification techniques include reverse-transcriptase PCR (RT-PCR), which is an accurate method for detecting viral RNA with an overall sensitivity of 73% and specificity of 99%.^51^ RT-PCR with primers directed against several genome sequences was developed for the detection of RSV while trying to achieve the highest sensitivity.

Multiplex quantitative real-time PCR (MRT-PCR) is rapid, accurate, and specific in detecting multiple viral pathogens of lower respiratory tract infection (LRTI), including RSV, with total sensitivity and specificity of 93.01% and 98.69%, respectively.^50^ The ultra-rapid real-time RT-PCR could detect RSV viral RNA in less than 20 minutes while maintaining sensitivity and specificity comparable to conventional real-time RT-PCR using large installed instruments. Furthermore, combining ultra-rapid real-time RT-PCR with specific sample preparation kits designed for detecting nucleic acids reduced the total working time for detecting RSV from clinical specimens to less than 25 minutes.^52-54^ TaqMan RT-qPCR, which detects two genes, the nucleocapsid (N) gene and the fusion (F) gene, is more sensitive than conventional PCR for RSV.^55,56^

A multiplex recombinase polymerase amplification assay combined with CRISPR/Cas12a could detect RSV at levels as low as 104 copies/mL within 50 minutes without cross-reactivity with other related pathogens. The sensitivity and specificity for RSV detection were 98.1% and 100%, respectively.^57,58^

Another fast, simple, and sensitive test for detecting RSV is real-time quantitative loop-mediated isothermal amplification (RT-qLAMP) with 95.1% and 100% sensitivity and specificity, respectively.^59^ Reverse transcription strand invasion-based amplification (RT-SIBA) assay showed good sensitivity by detecting as few as ten copies of RSV RNA within 20 minutes. When investigated using nasopharyngeal swab specimens, it showed 100% sensitivity and specificity.^60^ Despite the excellent accuracy of nucleic acid amplification tests, they are not widely available, especially in developing healthcare systems, due to their cost.^61^

Other diagnostic techniques for RSV that rely on the detection of viral antigens are more widely available due to their lower cost; however, they have lower sensitivity compared to nucleic acid amplification rests; they include immunofluorescence (IF) techniques such as the rapid fluorescent immunochromatographic test (FICT)^62^ and direct fluorescent antibody test (DFA).^63^ Rapid antigen-detecting tests give a rapid qualitative positive or negative, and they include immunochromatographic (ICR) tests, enzyme immunoassays (EIA), and optical immunoassays (OIA).^64-67^ A 2018 study evaluated the radiological findings of RSV infection among 400 patients. The most common chest radiograph abnormalities were interstitial prominence, airspace opacity, and hyperinflation. Patients with severe clinical courses had higher airspace opacity than those with a non-severe course. Unilateral airspace opacity was more common than bilateral opacity, and hyperinflation was common in severe cases.^31^

## 8. Treatment

Supportive care is the primary focus of RSV treatment, with antiviral therapy playing a lesser role. Supportive care involves supplemental oxygen, MV, nutritional support, and fluid replacement therapy, and it may also require inhaled bronchodilators, inhaled hypertonic saline, and corticosteroids.^68-70^ The efficacy of corticosteroids in reducing symptoms and preventing complications of RSV infection lacks conclusive evidence.^71^ Some studies proposed that inhaled hypertonic saline may aid in airway mucus clearance, thus leading to a shorter hospital stay.^72^

### 8.1. Antiviral therapy

Antiviral therapy, particularly Ribavirin, a broad-spectrum guanosine analogue effective against many RNA viruses, is the only Food and Drug Administration (FDA)-approved drug for RSV treatment.^71,73,74^ Ribavirin is primarily utilized in immunocompromised patients. Studies found many benefits of inhaled Ribavirin, such as a decreased hospital length of stay and conversion of upper respiratory tract infection (URTI) to LRTI.^68-70^

Ribavirin exists in two forms: aerosolized (FDA-approved) and oral (under study). Many comparative studies explore their effectiveness, side effects, pricing, and administration ease. Some studies suggest that oral Ribavirin provides similar efficacy to inhaled Ribavirin at a lower cost.^73,75^

Research into newer antivirals reveals promising candidates like GS-5806 (presatovir), which inhibits the RSV envelope’s fusion with the host cell, and RV-521 (sisunatovir), which inhibits the RSV F protein. Both in phase 2 clinical trials, these agents show efficacy in reducing viral loads and symptoms.^68,76–78^ ALX-0171 is another experimental agent that was shown to accelerate the virus load decrease.^79^

## 9. Global Impact and Transmission Dynamics

RSV orchestrates its spread through a diverse repertoire of transmission routes. Like many other respiratory viruses, RSV utilizes large droplet inoculation as its primary route, requiring close contact with an infected individual or via contaminated surfaces. This intricate transmission unfolds primarily through four major modes: direct contact, indirect contact/fomite, droplet, and aerosol transmission.^23^ Direct contact involves transferring infectious viral particles from an infected individual to a susceptible host, often through coughing, sneezing, or close physical contact. This mode is particularly effective when infected individuals are symptomatic and actively shedding the virus.^24^

Indirect contact/fomite transmission, a subtle yet potent threat, lurks on inanimate surfaces. When an infected individual expels respiratory secretions, viral particles can land on objects like toys, doorknobs, or clothes. These contaminated surfaces become silent partners in the transmission chain, potentially infecting susceptible individuals who touch them.^80^ This underscores the need for cleaning and disinfecting children’s toys, especially in hospital environments.

Notably, RSV exhibits remarkable persistence on surfaces, remaining active for up to 5 hours on some materials. However, its longevity on the skin is considerably shorter, lasting only around 30 minutes, highlighting the importance of hand hygiene as a preventative measure.^24^

Droplet transmission occurs when infected individuals cough, sneeze, talk, or exhale. Large droplets, laden with viral particles, are propelled through the air, capable of infecting others within a short distance (typically within 1–2 meters).^81^ Unlike their larger counterparts, aerosolized particles, smaller in size, can remain airborne for extended periods and travel over greater distances, potentially exposing individuals beyond the immediate vicinity of the infected source. This airborne transmission mode is particularly relevant in enclosed spaces with poor ventilation.^82^ The choice of transmission route is not a static performance but rather a dynamic interplay between viral characteristics, environmental factors, and host susceptibility. Large droplets, while effective over short distances, tend to settle quickly, limiting their reach. Conversely, smaller aerosols, though capable of longer-range travel, are less likely to deposit viral particles sufficiently to initiate infection. This intricate interplay between droplet size and distance^83^ emphasizes the importance of maintaining physical distancing and practicing proper respiratory hygiene to mitigate RSV transmission.

## 10. Vaccination and Prevention

Immunization against RSV can be done via passive immunization with monoclonal antibodies or active immunization via vaccines.

### 10.1. Monoclonal antibody immunization

In the 1990s, the first monoclonal antibody against the F protein of RSV, palivizumab, was developed and demonstrated to effectively reduce the severity of RSV infection when administered in high titers. Palivizumab is the most widely used prophylaxis for preventing RSV disease in infants. However, its high cost and the necessity for monthly dosing impose a substantial economic burden, particularly on low- and middle-income families.^84,85^

Another monoclonal antibody, motavizumab, showed promise in reducing the rates of RSV infection, RSV-related hospitalization, ICU admission, supplemental oxygen use, and MV.^85^ Meanwhile, palivizumab demonstrated efficacy mainly in reducing ICU admissions. Motavizumab’s association with skin hypersensitivity reactions in recipients led to the FDA withholding licensure.^85,86^

Nirsevimab represents a significant advancement. It is an extended half-life monoclonal antibody administered in a single dose. It is as efficacious as palivizumab and more cost-effective.^84,87,88^ Nirsevimab received FDA approval in 2023, offering a more convenient and potentially economical option for RSV prevention.

Clesrovimab, an extended half-life monoclonal antibody, is currently under clinical development and in a phase III trial.^84,88^ The outcome of this trial will determine its potential as a future addition to the armamentarium of monoclonal antibodies against RSV, expanding the options available for prevention and treatment.

The local administration of monoclonal antibodies presents a promising avenue and holds the potential for substantial cost reduction through dose minimization. Nevertheless, the evidence for the efficacy of local administration of monoclonal antibodies in preventing RSV infection is lacking currently.^88,89^

As we mentioned, RSV F and G proteins are responsible for viral attachment to cells, and thus, neutralizing antibodies against these proteins provide protective immunity.^13,90^ Protein F is the target of the available vaccines and monoclonal antibodies.^91^ This preference stems from the genetic stability and conservation of protein F across RSV genotypes, unlike the more variable protein G. One study found that palivizumab is more effective at suppressing RSV-A groups than RSV-B groups.^13,90^

It is critical to step up research efforts to create locally administered antibodies that can successfully fend off RSV infection. The focus should be on subtype-specific immunity in creating next-generation antibodies, reflecting a critical imperative in pursuing enhanced RSV protection.

### 10.2. Vaccination

Immunity to RSV is incomplete, leading to reinfections throughout an individual’s life.^92^ Initial attempts to develop an RSV vaccine date back to the 1960s, with an unsuccessful formalin-inactivated RSV vaccine that induced vaccine-associated enhanced respiratory disease via antibody-mediated enhancement. Subsequent efforts resulted in the development of 24 vaccines against RSV, two of which, Arexvy by GlaxoSmithKline Biologicals (GSK) and Abrysvo by Pfizer, have received FDA approval for market use as preventive agents against lower respiratory tract infections. Most of these vaccines target the F protein of RSV.^91,92^

These RSV vaccines utilize various approaches such as mRNA, subunit and particle-based, live-attenuated or chimeric vaccines, and recombinant vector-based vaccines. Arexvy is identified as a single-dose subunit vaccine, while Abrysvo is classified as a single-dose bivalent subunit vaccine. Both vaccines demonstrated favorable safety profiles and efficacy.^91,92^

The utilization of mRNA in vaccine development represents a significant breakthrough, providing high efficacy against SARS-CoV-2 infection and adaptability for diverse viruses.^88,93^ A novel mRNA-based RSV vaccine, mRNA-1345, encoding the stabilized RSV prefusion F glycoprotein, is currently under Phase III of clinical development, demonstrating an efficacy of 83.7% in preventing lower respiratory tract disease.^94^

As per the CDC recommendations, adults aged 60 and older are advised to receive a single dose of either Arexvy or Abrysvo. Abrysvo is specifically recommended for pregnant mothers during the first 32–36 weeks of pregnancy. Additionally, the administration of Nirsevimab is recommended for infants and certain young children who are at an increased risk of severe RSV disease.^95^

## 11. COVID-19 Effect

The influence of nonpharmaceutical interventions associated with COVID-19 on the seasonality of RSV and other respiratory infections was remarkable. In the first year of the COVID-19 pandemic, RSV cases globally declined rapidly.^96,97^ However, following this decline, numerous regions worldwide observed varying degrees of off-season resurgence in RSV cases. Interestingly, there was significant interregional variability in the intensity and clinical severity of the resurgence.^97^ Additionally, certain countries reported a notable shift in affected demographics, with a growing number of cases observed in older children.^96^

One plausible interpretation of these trends in the context of the COVID-19 pandemic is the prolonged lack of physical contact and viral exposure which contributed to an increased pool of immune-naive children, potentially rendering them more susceptible to RSV and other respiratory infections.^96,97^ Moreover, speculation arose regarding a potential interaction between SARS-CoV-2 and RSV and the dominance of virulent RSV strains.^97,98^ However, it is crucial to note that, as of now, no strong evidence supports such an interaction. It is worth mentioning that COVID-19 infection may have induced immune dysregulation that extends beyond the recovery period.^97,99^

## 12. RSV as Oncolytic Virus

Oncolytic viruses possess the unique ability to infect cancer cells while leaving normal cells unharmed. These viruses, either occurring naturally or engineered through genetic modifications, are tailored to replicate specifically within cancer cells. By doing so, they induce cell lysis within the tumor while preserving the integrity of surrounding healthy tissue.^100^ In the case of the RSV-A2 strain, there is substantial evidence supporting its oncolytic function. Investigations into the oncolytic capabilities of RSV-A2 have been conducted across various tumor tissues, including the skin^101^, hepatocellular carcinoma cells^102^, breast, cervical,^100^ and prostate^103^ cancer cells. RSV-A2 demonstrates its oncolytic potential by inducing apoptosis.^100^ This targeted approach holds promise for developing novel and precise therapies in cancer treatment.

## 13. Conclusion

RSV remains a significant global health challenge, particularly impacting children under the age of five and the elderly. Understanding its taxonomy, virion structure, genome, and proteins provides crucial insights into its pathogenesis, transmission dynamics, and clinical manifestations. The variability in RSV strains and their dominance underscores the challenges in developing effective vaccines and therapies.

Diagnostic techniques, particularly nucleic acid amplification tests, have significantly improved RSV detection, although accessibility remains a challenge, especially in low-resource settings. Supportive care remains fundamental in managing RSV infections, with antiviral therapy playing a limited role.

Preventive strategies, including monoclonal antibody immunization and vaccination, offer promising avenues for mitigating RSV’s impact. The recent FDA approvals of Nirsevimab and RSV vaccines represent significant strides in RSV prevention. Notably, promising efficacy rates have been demonstrated by mRNA technology in vaccine development. Additionally, exploring RSV’s oncolytic potential offers intriguing prospects for cancer treatment, showcasing its versatility beyond respiratory infections. Overall, sustained research efforts and global collaboration are indispensable in effectively combating RSV and alleviating its burden on public health.

## Authors’ Contributions

All authors contributed to the literature review, writing, and manuscript review.

## Disclosures

The authors have no conflicts of interest to disclose.
